# Analysis of Blind Reconstruction of BCH Codes

**DOI:** 10.3390/e22111256

**Published:** 2020-11-05

**Authors:** Soonhee Kwon, Dong-Joon Shin

**Affiliations:** Department of Electronics and Computer Engineering, Hanyang University, Seoul 04763, Korea; tnsgml1991@hanyang.ac.kr

**Keywords:** blind reconstruction, BCH codes, galois field, galois field fourier transform, lower-bound, RS codes

## Abstract

In this paper, the theoretical lower-bound on the success probability of blind reconstruction of Bose–Chaudhuri–Hocquenghem (BCH) codes is derived. In particular, the blind reconstruction method of BCH codes based on the consecutive roots of generator polynomials is mainly analyzed because this method shows the best blind reconstruction performance. In order to derive a performance lower-bound, the theoretical analysis of BCH codes on the aspects of blind reconstruction is performed. Furthermore, the analysis results can be applied not only to the binary BCH codes but also to the non-binary BCH codes including Reed–Solomon (RS) codes. By comparing the derived lower-bound with the simulation results, it is confirmed that the success probability of the blind reconstruction of BCH codes based on the consecutive roots of generator polynomials is well bounded by the proposed lower-bound.

## 1. Introduction

In order to achieve reliable information transmission through noisy communication channels, the use of error-correcting codes (ECCs) in data-stream is indispensable [1]. By sharing the parameters of ECCs between the transmitter and the receiver, the errors occurred by communication channels can be detected or corrected at the receiver in a cooperative way. However, in a non-cooperative context, it is necessary to decode received (or intercepted) data without the knowledge of parameters of the used ECC. In other words, a blind reconstruction of the parameters of the used ECC should be performed by the receiver.

A blind reconstruction of ECCs has been studied in various ways [2,3,4,5,6,7,8,9,10,11,12,13,14,15,16,17,18,19]. The blind reconstruction schemes of linear block codes are studied in [2,3,4,5,6,7,8,9], the blind reconstruction schemes of Bose–Chaudhuri–Hocquenghem (BCH) codes are studied in [10,11,12,13,14,15], and the blind reconstruction schemes of convolutional codes are studied in [16,17,18,19]. Most of the blind reconstruction schemes of ECCs take the dual code approach to reconstruct the dual code space of the used code by using the received codewords. Valembois [2] proposed a detection and recognition algorithm for binary linear codes by using the dual code property and Cluzeau [3] proposed a blind reconstruction method based on iterative decoding techniques by using the dual code property. Moreover, most of the blind detection methods of BCH codes are also based on the dual code approach. By using the properties of BCH codes, their parity-check matrices can be constructed through applying Galois field Fourier transform (GFFT) on the received codewords and many of the blind reconstruction methods of BCH codes are based on GFFT [10,11,12,13,14,15]. In the same manner, most of the blind reconstruction methods of convolutional codes are also based on the dual code approach [16,17,18,19]. To reconstruct the generator polynomial or generator matrix of convolutional code, its dual code is recognized preferentially.

An analysis of the blind reconstruction of cyclic codes over binary erasure channel (BEC) is performed in [20]. Note that for BEC, the number and the locations of error bits in the received data-stream are known to the receiver. By using this property of BEC, a blind reconstruction scheme of binary cyclic codes is proposed and a lower-bound on the detection probability of this scheme is analyzed in [20]. However, many blind reconstruction schemes consider the binary symmetric channel (BSC) where the number and the locations of error bits in the received data-stream are not unavailable. Therefore, the analysis in [20] is not directly applicable to the blind reconstruction schemes considering the BSC.

In this paper, the blind reconstruction of BCH codes over *q*-ary symmetric channel is mainly considered because BCH codes are a most widely used class of cyclic codes, especially in communication and storage systems and *q*-ary symmetric channel is a general form of BSC. Especially, the method in [15] shows the best blind reconstruction performance among the existing blind reconstruction methods of BCH codes, but the theoretical analysis of this method has not been performed yet. Therefore, by analyzing the properties of BCH codes on the aspects of blind reconstruction, a lower-bound on the success probability of the blind reconstruction method in [15] is derived. More specifically, the distribution of GFFT values of the received codewords is analyzed and the blind reconstruction method is formulated by using the conjugacy classes. By comparing the derived lower-bound with the simulation results, it is confirmed that the success probability of the blind reconstruction is well lower-bounded. Furthermore, the analysis of BCH codes on the aspects of blind reconstruction may lay a foundation for an analysis of other blind reconstruction methods of BCH codes based on GFFT.

In Section 2, definitions and properties of BCH codes and GFFT are briefly explained. In Section 3, the theoretic analysis of the properties of BCH codes on the aspects of blind reconstruction is performed. In Section 4, the blind reconstruction method in [15] is explained, and a lower-bound on the success probability of this blind reconstruction method is derived. The simulation results confirm that the success probability of the blind reconstruction method is well-bounded by the derived lower-bound. In Section 5, conclusions are provided.

## 2. BCH Codes and Galois Field Fourier Transform

In this section, the BCH codes and the Galois field Fourier transform (GFFT) are briefly described.

### 2.1. BCH Codes

BCH codes is a class of linear block codes for forward error correction. Let GF(q) denote the Galois field (or finite field) of *q* elements and let $BCH_{q}\left(n,k\right)$ denote the BCH code with length *n* and dimension *k* over GF(q). Note that the dimension *k* is the same as the length of random message which also implies the number of codewords. Then, the generator polynomial of $BCH_{q}\left(n,k\right)$ is defined as follows:

(1) $$g\left(x\right)=LCM[M_{\alpha^{b}}\left(x\right),M_{\alpha^{b+1}}\left(x\right),\cdots ,M_{\alpha^{b+d-2}}\left(x\right)]$$

where LCM denotes the least common multiple function, α is a primitive *n*-th root of unity in $GF(q^{m})$, $M_{\alpha^{i}}\left(x\right)$ is a minimal polynomial of $\alpha^{i}$ over GF(q), *b* is an arbitrary positive integer smaller than *n*, and *d* is a designed distance. Note that *m* is the smallest integer such that *n* divides $q^{m}-1$. By the definition of generator polynomial g(x), $\alpha^{b}$, $\alpha^{b+1}$, ⋯, $\alpha^{b+d-2}$ are the roots of g(x), i.e., $g\left(\alpha^{b}\right)=g\left(\alpha^{b+1}\right)=\cdots =g\left(\alpha^{b+d-2}\right)=0$. Let $S_{r}$ be the set of the exponents of all roots of g(x) as follows:

(2) $$S_{r}=\left\{i|g\left(\alpha^{i}\right)=0,i\in \left\{0,1,\cdots ,n-1\right\}\right\}.$$

A message can be expressed in polynomial form as $m\left(x\right)=m_{0}+m_{1}x+\cdots +m_{k-1}x^{k-1}$ and in vector form as $m=(m_{0},m_{1},\cdots ,m_{k-1})$, where $m_{i}\in GF\left(q\right)$ for i∈{0,1,⋯,k−1}. A codeword of $BCH_{q}\left(n,k\right)$ can be expressed in polynomial form as $c\left(x\right)=c_{0}+c_{1}x+\cdots +c_{n-1}x^{n-1}$ and in vector form as $c=(c_{0},c_{1},\cdots ,c_{n-1})$, where $c_{i}\in GF\left(q\right)$ for i∈{0,1,⋯,n−1}. Then, c(x) can be obtained as follows:

(3) c(x)=m(x)g(x).

Since a codeword c(x) has g(x) as a factor, all roots of g(x) are also roots of c(x), i.e., $c(\alpha^{i})=0$ for all $i\in S_{r}$. In this paper, the *q*-ary symmetric channel with error probability ϵ is considered. Channel error can be expressed in polynomial form as $e\left(x\right)=e_{0}+e_{1}x+\cdots +e_{n-1}x^{n-1}$ and in vector form as $e=(e_{0},e_{1},\cdots ,e_{n-1})$, where $e_{i}\in GF\left(q\right)$ for i∈{0,1,⋯,n−1}. Note that by the definition of *q*-ary symmetric channel, $\mathrm{Pr}(e_{i}=0)=1-\epsilon$ and $\mathrm{Pr}\left(e_{i}=x\right)=\epsilon /\left(q-1\right)$ for i∈{0,1,⋯,n−1} and $x\in GF^{*}\left(q\right)$ where $GF^{*}\left(q\right)=GF\left(q\right)\setminus \left\{0\right\}$. Then, a received codeword at the receiver is expressed in polynomial form as

(4) r(x)=c(x)+e(x),

or in vector form as follows:

(5) r=c+e.

Throughout the paper, the polynomial form and the vector form will be used interchangeably.

If there is no error (i.e., e(x)=0), $r(\alpha^{i})=0$ for all $i\in S_{r}$ because r(x)=c(x). However, if e(x)≠0, we may have $r(\alpha^{i})\neq 0$ for some $i\in S_{r}$ because it can be $e(\alpha^{i})\neq 0$ for some $i\in S_{r}$.

### 2.2. Conjugacy Classes and Cyclotomic Cosets

Let $U_{\beta }$ denote a conjugacy class of $\beta \in GF(q^{m})$. Then, $U_{\beta }$ consists of β and its conjugates $\beta^{q}$, $\beta^{q^{2}}$, $\beta^{q^{3}}$, ⋯. Note that the conjugacy classes of the elements in the same conjugacy class are the same. The minimal polynomial of $\alpha^{i}\in GF\left(q^{m}\right)$ over GF(q), $M_{\alpha^{i}}\left(x\right)$, can be obtained by using the conjugacy classes as follows:

(6) $$M_{\alpha^{i}}\left(x\right)=\prod_{z\in U_{\alpha^{i}}}\left(x-z\right).$$

The degree of $M_{\alpha^{i}}\left(x\right)$ is equal to $|U_{\alpha^{i}}|$, where |S| is the cardinality of a set *S*. Note that since $M_{\alpha^{i}}\left(x\right)$ has all the elements in $U_{\alpha^{i}}$ as its roots, g(x) in (1) has all the elements in $U_{\alpha^{b}}$, $U_{\alpha^{b+1}}$, ⋯, $U_{\alpha^{b+d-2}}$ as its roots. Let $S_{N}$ denote the null spectrum of the $BCH_{q}\left(n,k\right)$ which has the generator polynomial in (1). Then $S_{N}$ is obtained as follows:

(7) $$S_{N}=\overset{b+d-2}{\underset{i=b}{⋃}}U_{\alpha^{i}}.$$

$S_{r}$ in (2) is also expressed as the set of the exponents of the elements in $S_{N}$ such as $S_{r}=\left\{i|\alpha^{i}\in S_{N}\right\}$. Then, the complement of $S_{r}$, denoted by ${S_{r}}^{c}$, is obtained as follows:

(8) $${S_{r}}^{c}=\left\{i|\alpha^{i}\in GF^{*}\left(q^{m}\right)\setminus S_{N}\right\}.$$

It is clear that ${S_{r}}^{c}=Z_{n}\setminus S_{r}$ where $Z_{n}=\left\{0,1,\cdots ,n-1\right\}$.

Let $C_{i}$ denote the cyclotomic coset of *i* modulo *n* with respect to GF(q). Then, the exponents of all the elements in $U_{\alpha^{i}}$ make up $C_{i}$ and $S_{r}={⋃}_{i=b}^{b+d-2}C_{i}$ by (2) and (7).

### 2.3. Galois Field Fourier Transform

The roots of a received codeword r(x) can also be obtained by performing the Galois field Fourier transform (GFFT) on r(x). The GFFT of c(x), denoted as C(X), can be expressed in polynomial form as follows:

(9) $$C\left(X\right)=c\left(\alpha^{0}\right)+c\left(\alpha^{1}\right)X+\cdots +c\left(\alpha^{n-1}\right)X^{n-1},$$

where $c\left(\alpha^{i}\right)\in GF\left(q^{m}\right)$ for i∈{0,1,⋯,n−1}. It is also expressed in vector form as follows:

(10) $$C=\left(C_{0},C_{1},\cdots ,C_{n-1}\right)=\left(c\left(\alpha^{0}\right),c\left(\alpha^{1}\right),\cdots ,c\left(\alpha^{n-1}\right)\right).$$

The GFFT matrix $M_{G}$ is defined as follows:

(11) $$M_{G}=\left[\begin{array}{ccccc} \alpha^{0} & \alpha^{0} & \alpha^{0} & \cdots & \alpha^{0}\\ \alpha^{0} & \alpha^{1} & \alpha^{2} & \cdots & \alpha^{n-1}\\ \alpha^{0} & \alpha^{2} & \alpha^{4} & \cdots & \alpha^{2(n-1)}\\ \vdots & \vdots & \vdots & \ddots & \vdots \\ \alpha^{0} & \alpha^{n-1} & \alpha^{2(n-1)} & \cdots & \alpha^{{\left(n-1\right)}^{2}} \end{array}\right].$$

Then, the GFFT of c is simply obtained by $C=c\times M_{G}$. By the definition of g(x) in (1), $C_{b}=C_{b+1}=\cdots =C_{b+d-2}=0$.

The GFFT of r(x), denoted as R(X), can be expressed in polynomial form as follows:

(12) $$R\left(X\right)=r\left(\alpha^{0}\right)+r\left(\alpha^{1}\right)X+\cdots +r\left(\alpha^{n-1}\right)X^{n-1},$$

where $r\left(\alpha^{i}\right)\in GF\left(q^{m}\right)$ for i∈{0,1,⋯,n−1}. The vector form of R(X) is expressed as follows:

(13) $$R=\left(R_{0},R_{1},\cdots ,R_{n-1}\right)=\left(r\left(\alpha^{0}\right),r\left(\alpha^{1}\right),\cdots ,r\left(\alpha^{n-1}\right)\right).$$

By using $M_{G}$ in (11), the GFFT of r is simply obtained by $R=r\times M_{G}$. In the error-free case (i.e., e(x)=0), $R_{b}=R_{b+1}=\cdots =R_{b+d-2}=0$. However, if e(x)≠0, we may have $R_{i}\neq 0$ for some i∈{b,b+1,⋯,b+d−2}.

The GFFT of e(x), denoted as E(X), can be expressed in polynomial form as follows:

(14) $$E\left(X\right)=e\left(\alpha^{0}\right)+e\left(\alpha^{1}\right)X+\cdots +e\left(\alpha^{n-1}\right)X^{n-1},$$

where $e\left(\alpha^{i}\right)\in GF\left(q^{m}\right)$ for i∈{0,1,⋯,n−1}. The vector form of E(X) is expressed as follows:

(15) $$E=\left(E_{0},E_{1},\cdots ,E_{n-1}\right)=\left(e\left(\alpha^{0}\right),e\left(\alpha^{1}\right),\cdots ,e\left(\alpha^{n-1}\right)\right).$$

By using (5), (10) and (13), it is clear that $R=C+E=\left(c+e\right)M_{G}$.

## 3. Theoretical Analysis of BCH Codes on the Aspects of Blind Reconstruction

### 3.1. GFFT of a Single Symbol Error

In this subsection, the GFFT values of a single symbol error is investigated. Let wt(a) denote the Hamming weight of a vector a, i.e., wt(a) is the number of non-zero elements in a. Note that a single symbol error e(x) satisfies wt(e)=1.

**Lemma** **1.** *If a received codeword r(x) of $BCH_{q}\left(n,k\right)$ contains a single symbol error, then*

(16) $$r\left(\alpha^{i}\right)\neq 0,\forall i\in S_{r}.$$

**Proof.**  Let $e\left(x\right)=e_{j}x^{j}$ for some j∈{0,1,⋯,n−1} and $e_{j}\in GF^{*}\left(q\right)$. Since the GFFT value of e(x) with respect to $\alpha^{i}$ is $E_{i}=e\left(\alpha^{i}\right)=e_{j}\alpha^{ij}\neq 0$ for all i∈{0,1,⋯,n−1} and $C_{i}=0$ for $i\in S_{r}$, $R_{i}=C_{i}+E_{i}\neq 0$ for $i\in S_{r}$. □

Lemma 1 shows that if r(x) contains a single symbol error, any root of g(x) cannot be a root of r(x). In the next subsection, the distribution of GFFT values of c(x) is analyzed.

### 3.2. GFFT of Codewords

Let $S_{c(\alpha^{i})}$ denote the set of the GFFT values taken by all the codewords c(x) of $BCH_{q}\left(n,k\right)$ for $x=\alpha^{i}$ as follows:

(17) $$S_{c(\alpha^{i})}≜\left\{c\left(\alpha^{i}\right)|c\left(x\right)\in BCH_{q}\left(n,k\right)\right\}.$$

Suppose that the minimal polynomial $M_{\alpha }\left(x\right)$ of a primitive *n*-th root of unity $\alpha \in GF(q^{m})$ over GF(q) has a degree $m^{\prime }$ where $m^{\prime }|m$. Then, any $\alpha^{i}\in GF\left(q^{m}\right)$ can be expressed by a linear combination of $\alpha^{0},\alpha^{1},\cdots ,\alpha^{m^{\prime }-1}$ as follows:

(18) $$\alpha^{i}=h_{0}+h_{1}\alpha +\cdots +h_{m^{\prime }-1}\alpha^{m^{\prime }-1},$$

where $h_{i}\in GF\left(q\right)$ for $i\in \{0,1,\cdots ,m^{\prime }-1\}$. Moreover, based on (18), any $\alpha^{i}\in GF\left(q^{m}\right)$ can be expressed in vector form, denoted as $v_{\alpha^{i}}$, as follows:

(19) $$v_{\alpha^{i}}=\left(h_{0},h_{1},\cdots ,h_{m^{\prime }-1}\right).$$

Note that $v_{\alpha^{i}}$ is a row vector. Let $V_{\alpha^{i}}\in GF{\left(q\right)}^{n\times m^{\prime }}$ be a matrix with $v_{{\left(\alpha^{i}\right)}^{0}}$, $v_{{\left(\alpha^{i}\right)}^{1}}$, ⋯, $v_{{\left(\alpha^{i}\right)}^{n-1}}$ as its rows, and $rk(\alpha^{i})$ denote the rank of $V_{\alpha^{i}}$ over GF(q).

**Lemma** **2.** *Suppose that a message m(x) is generated uniformly at random, a codeword c(x) of $BCH_{q}\left(n,k\right)$ is encoded by g(x) as in (3), and $k\geq rk(\alpha^{i})$. Then, it is satisfied that*

(20) $$S_{c(\alpha^{i})}=\left\{0\right\},\forall i\in S_{r},$$

(21) $$|S_{c(\alpha^{i})}|=q^{rk(\alpha^{i})},\forall i\in {S_{r}}^{c},$$

(22) $$\mathrm{Pr}\left(c(\alpha^{i})=x\right)=\frac{1}{|S_{c(\alpha^{i})}|},\forall x\in S_{c(\alpha^{i})}.$$

**Proof.**  First of all, for any $i\in S_{r}$, it is always true that $c(\alpha^{i})=0$ due to the definition of $S_{r}$. Therefore, $S_{c(\alpha^{i})}=\left\{0\right\}$ and $\mathrm{Pr}\left(c\left(\alpha^{i}\right)=0\right)=1/\left|S_{c(\alpha^{i})}\right|=1$ for any $i\in S_{r}$.

Second, in order to prove (21), let $\Gamma \in GF{\left(q\right)}^{q^{k}\times n}$ denote a matrix having all the $q^{k}$ codewords of $BCH_{q}\left(n,k\right)$ as its rows. Then, the GFFT values of $q^{k}$ codewords can be expressed in vector form as follows:

(23) $$\Lambda =\Gamma \times V_{\alpha^{i}},$$

where $\Lambda \in GF{\left(q\right)}^{q^{k}\times m^{\prime }}$ is a matrix with the vector forms of all GFFT values of $q^{k}$ codewords with respect to $\alpha^{i}$ as its rows. Note that the rank of Γ is *k* because all the rows of Γ are the codewords of $BCH_{q}\left(n,k\right)$, and the rank of $V_{\alpha^{i}}$ is $rk(\alpha^{i})$ by the definition. The matrix Γ can be decomposed as Γ=Δ×G where Δ has all the elements of $GF{\left(q\right)}^{k}$ as its rows and G is the generator matrix of $BCH_{q}\left(n,k\right)$. Note that the rank of Λ, rank(Λ), is equal to $rank\left(\Gamma \times V_{\alpha^{i}}\right)=rank\left(\Delta \times G\times V_{\alpha^{i}}\right)$. Since the size of Δ is $q^{k}\times k$ and rank(Δ)=k, $rank\left(\Delta \times G\times V_{\alpha^{i}}\right)=rank\left(G\times V_{\alpha^{i}}\right)$. The *j*-th row of $G\times V_{\alpha^{i}}$ is expressed as $g_{j}\times V_{\alpha^{i}}$ where $g_{j}$ is the *j*-th row of G, j∈{1,2,⋯,k}. Since $g_{j}\times V_{\alpha^{i}}\neq 0$ for $i\in {S_{r}}^{c}$ and j∈{1,2,⋯,k}, $g_{1},g_{2},\cdots ,g_{k}$ are linearly independent, and $k\geq rk(\alpha^{i})$, it is clear that $rank(G\times V_{\alpha^{i}})$ is equal to $rk(\alpha^{i})$. Therefore, the rank of Λ is also equal to $rk(\alpha^{i})$, which implies that there are $q^{rk(\alpha^{i})}$ distinct rows in Λ and $|S_{c(\alpha^{i})}|=q^{rk(\alpha^{i})}$ for any $i\in {S_{r}}^{c}$.

Lastly, in order to show (22), let $x_{0},x_{1},\cdots ,x_{n_{1}-1}\in GF{\left(q\right)}^{n}$ be all distinct codewords such that $x_{0}\left(\alpha^{i}\right)=x_{1}\left(\alpha^{i}\right)=\cdots =x_{n_{1}-1}\left(\alpha^{i}\right)=x$ for given *i* and $x\in GF(q^{m})$. Also, let $y_{0},y_{1},\cdots ,y_{n_{2}-1}\in GF{\left(q\right)}^{n}$ be all distinct codewords such that $y_{0}\left(\alpha^{i}\right)=y_{1}\left(\alpha^{i}\right)=\cdots =y_{n_{2}-1}\left(\alpha^{i}\right)=y$ for the same *i* and $y\in GF(q^{m})$. These relations can be expressed in matrix multiplication as follows:

(24) $$\left[\begin{array}{c} x_{0}\\ x_{1}\\ \vdots \\ x_{n_{1}-1} \end{array}\right]\times M_{G}^{\left(i+1\right)}=\left[\begin{array}{c} x\\ x\\ \vdots \\ x \end{array}\right],$$

(25) $$\left[\begin{array}{c} y_{0}\\ y_{1}\\ \vdots \\ y_{n_{2}-1} \end{array}\right]\times M_{G}^{\left(i+1\right)}=\left[\begin{array}{c} y\\ y\\ \vdots \\ y \end{array}\right],$$

where $M_{G}^{\left(i+1\right)}$ is the (i+1)-st column of $M_{G}$ in (11). In order to show $\mathrm{Pr}\left(c\left(\alpha^{i}\right)=x\right)=1/\left|S_{c(\alpha^{i})}\right|$, it is enough to show $n_{1}=n_{2}$. Without loss of generality, suppose that $n_{1}>n_{2}$. From (24), we can obtain

(26) $$\left[\begin{array}{c} x_{0}-x_{0}\\ x_{1}-x_{0}\\ \vdots \\ x_{n_{1}-1}-x_{0} \end{array}\right]\times M_{G}^{\left(i+1\right)}=\left[\begin{array}{c} 0\\ 0\\ \vdots \\ 0 \end{array}\right].$$

Note that $n_{1}$ vectors $x_{i}-x_{0}$ are all distinct. By adding $y_{0}$ to each row of the first matrix in LHS of (26), we obtain

(27) $$\left[\begin{array}{c} y_{0}+x_{0}-x_{0}\\ y_{0}+x_{1}-x_{0}\\ \vdots \\ y_{0}+x_{n_{1}-1}-x_{0} \end{array}\right]\times M_{G}^{\left(i+1\right)}=\left[\begin{array}{c} y\\ y\\ \vdots \\ y \end{array}\right].$$

Note that $n_{1}$ vectors $y_{0}+x_{i}-x_{0}$ are still all distinct and they are valid codewords. According to (27), the number of codewords which have *y* as the GFFT value with respect to $\alpha^{i}$ is $n_{1}$, which is a contradiction to the assumption $n_{1}>n_{2}$ and hence $n_{1}=n_{2}$. Therefore, if GFFT is performed on all the codewords of $BCH_{q}\left(n,k\right)$ with respect to $\alpha^{i}$, all the elements of $S_{c(\alpha^{i})}$ occur uniformly at random for the random message m(x), which implies $\mathrm{Pr}\left(c\left(\alpha^{i}\right)=x\right)=1/\left|S_{c(\alpha^{i})}\right|$ for any $i\in {S_{r}}^{c}$. □

By Lemma 2, it is clear that $c(\alpha^{i})=0$ for $i\in S_{r}$ and $c(\alpha^{i})$ for $i\in {S_{r}}^{c}$ takes a value from $S_{c\left(\alpha^{i}\right)}$ uniformly at random. In the next subsection, the distribution of GFFT values of r(x) is analyzed.

### 3.3. GFFT of Received Codewords

Consider a received codeword r(x)=c(x)+e(x) having a single symbol error, i.e., $e\left(x\right)=e_{j}x^{j}$ with $e_{j}\neq 0$. Let $S_{e(\alpha^{i})}$ be the set of all GFFT values of a single symbol error e(x) with respect to $\alpha^{i}$ as follows:

(28) $$\begin{array}{c} S_{e(\alpha^{i})}≜\left\{e\left(\alpha^{i}\right)|e\left(x\right)=e_{j}x^{j},e_{j}\in GF^{*}\left(q\right),j\in \left\{0,1,\cdots ,n-1\right\}\right\}. \end{array}$$

By using Lemma 2, the distribution of GFFT values of r(x) with a single symbol error is analyzed as follows.

**Corollary** **1.** *Suppose that a message m(x) is generated uniformly at random, a codeword c(x) of $BCH_{q}\left(n,k\right)$ is encoded by g(x) as (3), e(x) is a single symbol error, and $k\geq rk(\alpha^{i})$. Then, it is satisfied that*

(29) $$S_{e(\alpha^{i})}\subset S_{c(\alpha^{i})},\forall i\in {S_{r}}^{c}.$$

**Proof.**  By Lemma 2, if $k\geq rk(\alpha^{i})$, it is clear that $|S_{c(\alpha^{i})}|=q^{rk(\alpha^{i})}$ for $i\in {S_{r}}^{c}$, which means that $S_{c(\alpha^{i})}$ contains all the linear combinations of ${\left(\alpha^{i}\right)}^{0}$, ${\left(\alpha^{i}\right)}^{1}$, ⋯, ${\left(\alpha^{i}\right)}^{n-1}$ over GF(q). Therefore, $S_{c(\alpha^{i})}$ contains $S_{e(\alpha^{i})}$ for any $i\in {S_{r}}^{c}$ and (29) holds. □

Let $S_{r(\alpha^{i})}$ be the set of all GFFT values of r(x) with a single symbol error $e_{j}x^{j}$ with respect to $\alpha^{i}$ as follows:

(30) $$\begin{array}{c} S_{r(\alpha^{i})}≜\left\{r\left(\alpha^{i}\right)|r\left(x\right)=c\left(x\right)+e_{j}x^{j},e_{j}\in GF^{*}\left(q\right),c\left(x\right)\in BCH_{q}\left(n,k\right),j\in \left\{0,1,\cdots ,n-1\right\}\right\}. \end{array}$$

**Lemma** **3.** *Suppose that a message m(x) is generated uniformly at random, a codeword c(x) of $BCH_{q}\left(n,k\right)$ is encoded by g(x) as (3), e(x) is a single symbol error, and $k\geq rk(\alpha^{i})$. Then, it is satisfied that*

(31) $$S_{r(\alpha^{i})}=S_{c(\alpha^{i})},\forall i\in {S_{r}}^{c},$$

(32) $$\mathrm{Pr}\left(r(\alpha^{i})=x\right)=\frac{1}{q^{rk(\alpha^{i})}},\forall x\in S_{r(\alpha^{i})},\forall i\in {S_{r}}^{c}.$$

**Proof.**  Based on (30), $S_{r(\alpha^{i})}$ can be expressed as follows:

(33) $$\begin{array}{c} S_{r(\alpha^{i})}=\left\{c\left(\alpha^{i}\right)+e\left(\alpha^{i}\right)|c\left(\alpha^{i}\right)\in S_{c(\alpha^{i})},e\left(\alpha^{i}\right)\in S_{e(\alpha^{i})}\right\}. \end{array}$$

As shown in Corollary 1, if e(x) is a single symbol error and $k\geq rk(\alpha^{i})$, $S_{e(\alpha^{i})}\subset S_{c(\alpha^{i})}$ for any *i*∈${S_{r}}^{c}$. Therefore, $S_{r(\alpha^{i})}$ is equal to $S_{c(\alpha^{i})}$ for any $i\in {S_{r}}^{c}$.

The probability in (32) is derived as follows:

(34) $$\begin{array}{cc} \mathrm{Pr} & \left(r(\alpha^{i})=x\right)\\ \; & =\mathrm{Pr}\left(c\left(\alpha^{i}\right)+e\left(\alpha^{i}\right)=x\right)\\ \; & =\sum_{y\in S_{e(\alpha^{i})}}\mathrm{Pr}\left(c(\alpha^{i})=x-y\right)\mathrm{Pr}\left(e(\alpha^{i})=y\right)\\ \; & \underset{=}{\left(a\right)}\frac{1}{|S_{c(\alpha^{i})}|}\sum_{y\in S_{e(\alpha^{i})}}\mathrm{Pr}\left(e(\alpha^{i})=y\right)\\ \; & =\frac{1}{|S_{c(\alpha^{i})}|}\\ \; & =\frac{1}{q^{rk(\alpha^{i})}} \end{array}$$

for $x\in S_{r(\alpha^{i})}$ and $i\in {S_{r}}^{c}$. The equality (a) holds by Lemma 2. □

Lemma 3 assumes wt(e)=1, however, in practice, multiple errors also occur. If wt(e)>1, Lemma 1 does not hold because $r(\alpha^{i})$ can be 0 for some $i\in S_{r}$ even though e(x)≠0. Note that $\mathrm{Pr}(e(\alpha^{i})=0$, e(x)≠0, $i\in S_{r})$ is equal to the undetectable error probability of the BCH code which has $\left\{\alpha^{i}|i\in S_{r}\right\}$ as its null spectrum.

**Lemma** **4.** *Suppose that a message m(x) is generated uniformly at random, a codeword c(x) of $BCH_{q}\left(n,k\right)$ is encoded by g(x) as (3), e(x) is generated by q-ary symmetric channel with error probability ϵ, and $k\geq rk(\alpha^{i})$. Then, it is satisfied that*

(35) $$S_{r(\alpha^{i})}=S_{c(\alpha^{i})},\forall i\in {S_{r}}^{c},$$

(36) $$\mathrm{Pr}\left(r(\alpha^{i})=x\right)=\frac{1}{q^{rk(\alpha^{i})}},\forall x\in S_{r(\alpha^{i})},\forall i\in {S_{r}}^{c}.$$

**Proof.**  For $i\in {S_{r}}^{c}$, $S_{c(\alpha^{i})}$ contains all the linear combinations of ${\left(\alpha^{i}\right)}^{0}$, ${\left(\alpha^{i}\right)}^{1}$, ⋯, ${\left(\alpha^{i}\right)}^{n-1}$ over GF(q). Since the error e(x) is not a single symbol error anymore, $S_{e(\alpha^{i})}$ is defined as follows:

(37) $$S_{e(\alpha^{i})}=\left\{e\left(\alpha^{i}\right)\;|\;e\left(x\right)=\sum_{j=0}^{n-1}e_{j}x^{j},e_{j}\in GF\left(q\right)\right\}.$$

$S_{e(\alpha^{i})}$ also contains all the linear combinations of ${\left(\alpha^{i}\right)}^{0}$, ${\left(\alpha^{i}\right)}^{1}$, ⋯, ${\left(\alpha^{i}\right)}^{n-1}$ over GF(q) and hence $S_{r(\alpha^{i})}=S_{c(\alpha^{i})}$ for any $i\in {S_{r}}^{c}$ because $r\left(\alpha^{i}\right)=c\left(\alpha^{i}\right)+e\left(\alpha^{i}\right)$.

The probability in (36) is derived as follows:

(38) $$\begin{array}{cc} \mathrm{Pr} & \left(r(\alpha^{i})=x\right)\\ \; & =\mathrm{Pr}\left(c\left(\alpha^{i}\right)+e\left(\alpha^{i}\right)=x\right)\\ \; & =\sum_{y\in S_{e(\alpha^{i})}}\mathrm{Pr}\left(c(\alpha^{i})=x-y\right)\mathrm{Pr}\left(e(\alpha^{i})=y\right)\\ \; & =\frac{1}{|S_{c(\alpha^{i})}|}\sum_{y\in S_{e(\alpha^{i})}}\mathrm{Pr}\left(e(\alpha^{i})=y\right)\\ \; & =\frac{1}{|S_{c(\alpha^{i})}|}\\ \; & =\frac{1}{q^{rk(\alpha^{i})}} \end{array}$$

for $x\in S_{r(\alpha^{i})}$ and $i\in {S_{r}}^{c}$. □

As you can see from Lemmas 3 and 4, the conclusions (31) and (32) and (35) and (36) are the same. It implies that if the encoded message m(x) is generated uniformly at random, the GFFT of r(x) with respect to $\alpha^{i}$ takes a value in $S_{r(\alpha^{i})}$ uniformly at random regardless of the distribution of e(x) for $i\in {S_{r}}^{c}$. By Lemma 4, the probability that r(x) has $\alpha^{i}$ as its root for $i\in {S_{r}}^{c}$ is $1/q^{rk(\alpha^{i})}$. Based on Lemma 4, the performance of blind reconstruction method of BCH codes [15] is analyzed in the next section.

## 4. Analysis of Blind Reconstruction Method of BCH Codes

### 4.1. Blind Reconstruction Method of BCH Codes

In this subsection, the blind reconstruction method of BCH codes based on consecutive roots of generator polynomials [15] is described. In order to perform this method, it is assumed that the codeword synchronization is perfectly done and the code length *n* is known to the receiver. Suppose that *M* codewords are received. The *j*-th received codeword is expressed in polynomial form as $r_{j}\left(x\right)=r_{j,0}+r_{j,1}x+\cdots +r_{j,n-1}x^{n-1}$ and in vector form as $r_{j}=\left(r_{j,0},r_{j,1},\cdots ,r_{j,n-1}\right)$ for j∈{1,2,⋯,M}. Let $L_{j}$ denote the set of pairs consisting of the length *l* of the consecutive roots and the starting value *s* of these consecutive roots of $r_{j}\left(x\right)$ defined as follows:

(39) $$L_{j}≜\left\{\left(s,l\right)\;|\;s\in \left\{1,2,\cdots ,n-1\right\},2\leq l\in N,r_{j}\left(\alpha^{i}\right)=0,\forall i\in C_{s}^{l}\right\},$$

where $C_{s}^{l}≜\cup_{i=s}^{s+l-1}C_{i}$. For example, if $r_{j}=\left(0,0,1,0,1,1,0\right)$ is received, then the GFFT of $r_{j}$ is $R_{j}=\left(1,0,0,1,0,1,1\right)$, and therefore $L_{j}=\left\{\left(5,2\right)\right\}$. Note that 0<s<n and 2≤l of the elements in $L_{j}$. By using (39), for $r_{j}\left(x\right)$, the maximum length of consecutive roots (MLCR) $l_{j}^{max}$ and the corresponding starting value of consecutive roots (SVCR) $s_{j}^{max}$ are obtained as follows:

(40) $$\left(s_{j}^{max},l_{j}^{max}\right)=\arg\max_{\left(s_{j},l_{j}\right)\in L_{j}}l_{j}.$$

Let $S_{max}$ denote the set of $\left(s_{j}^{max},l_{j}^{max}\right)$ for j∈{1,2,⋯,M} as follows:

(41) $$S_{max}≜\left\{\left(s_{j}^{max},l_{j}^{max}\right)|j\in \left\{1,2,\cdots ,M\right\}\right\}$$

The blind reconstruction method of BCH codes in [15] has two-stage processes.

1. First stage: The most frequent $s_{j}^{max}$ in $S_{max}$ is selected and called a reference SVCR (R-SVCR), denoted as $s_{ref}$.
2. Second stage: The most frequent $l_{j}^{max}$ among the pairs having $s_{j}^{max}=s_{ref}$ in $S_{max}$ is selected and called a reference MLCR (R-MLCR), denoted as $l_{ref}$.

By setting $b=s_{ref}$ and $d=l_{ref}+1$ in (1), the generator polynomial of the used BCH code is reconstructed.

### 4.2. Performance Analysis of Blind Reconstruction Method of BCH Codes

In this subsection, the performance of blind reconstruction method in [15] is analyzed. Suppose that $BCH_{q}\left(n,k\right)$ is used and *M* codewords are received. The generator polynomial $g_{0}\left(x\right)$ is set as in (1) with $b=s_{0}$ and $d=l_{0}+1$. In order to succeed in blind reconstruction of this BCH code, $s_{ref}$ and $l_{ref}$ should be correctly determined as $s_{ref}=s_{0}$ and $l_{ref}=l_{0}$. Define the sets of received codewords, M(s,l), $M^{m}\left(s,l\right)$, $M^{*}\left(s,l\right)$, and $M^{e}\left(s,l\right)$ as follows:

(42) $$M\left(s,l\right)=\left\{r_{j}\left(x\right)|\left(s,l\right)\in L_{j}\right\},$$

(43) $$M^{m}\left(s,l\right)=\left\{r_{j}\left(x\right)|\left(s_{j}^{max},l_{j}^{max}\right)=\left(s,l\right)\right\},$$

(44) $$M^{*}\left(s,l\right)=\left\{r_{j}\left(x\right)\;|\;r_{j}\left(x\right)\in M^{m}\left(s,l\right),r_{j}\left(\alpha^{i}\right)\neq 0,\forall i\in \left\{1,2,\cdots ,n-1\right\}\setminus C_{s}^{l}\right\},$$

(45) $$M^{e}\left(s,l\right)=\left\{r_{j}\left(x\right)|e_{j}\left(x\right)\neq 0,r_{j}\left(x\right)\in M\left(s,l\right)\right\}.$$

Note that $M^{*}\left(s,l\right)\subseteq M^{m}\left(s,l\right)\subseteq M\left(s,l\right)$. In order to succeed in the first stage of the blind reconstruction method, the following relation should be satisfied,

(46) $$\sum_{l=2}^{n-1}|M^{m}\left(s_{0},l\right)|>\sum_{l=2}^{n-1}\left|M^{m}\left(s,l\right)\right|,\forall s\neq s_{0}.$$

The relation (46) can be simplified as in the following Lemma 5.

**Lemma** **5.** *If the following inequality is satisfied, then the first stage of the blind reconstruction of BCH codes in [15] always succeeds,*

(47) $$|M^{*}\left(s_{0},l_{0}\right)|>|M\left(s,2\right)|,\forall s\neq s_{0},$$

*and the success probability of the first stage is lower-bounded as*

(48) $$\mathrm{Pr}\left(\sum_{l=2}^{n-1}|M^{m}\left(s_{0},l\right)|>\sum_{l=2}^{n-1}\left|M^{m}\left(s,l\right)\right|,\forall s\neq s_{0}\right)\geq \mathrm{Pr}\left(|M^{*}\left(s_{0},l_{0}\right)|>|M\left(s,2\right)|,\forall s\neq s_{0}\right).$$

**Proof.**  In order to succeed in the first stage of the blind reconstruction method in [15], the relation (46) should be satisfied. The LHS in (46) satisfies the following inequalities,

(49) $$\begin{array}{cc} \sum_{l=2}^{n-1}\left|M^{m}\left(s_{0},l\right)\right| & \underset{\geq }{\left(a\right)}\sum_{l=2}^{n-1}\left|M^{*}\left(s_{0},l\right)\right|\\ \; & \underset{\geq }{\left(b\right)}|M^{*}\left(s_{0},l_{0}\right)|. \end{array}$$

The first inequality (a) is derived by using $M^{*}\left(s_{0},l\right)\subseteq M^{m}\left(s_{0},l\right)$, and the second inequality (b) is trivial. The RHS in (46) satisfies the following inequality,

(50) $$\begin{array}{cc} \sum_{l=2}^{n-1}\left|M^{m}\left(s,l\right)\right| & \underset{=}{\left(a\right)}|\overset{n-1}{\underset{l=2}{⋃}}M^{m}\left(s,l\right)|\\ \; & \underset{\leq }{\left(b\right)}|\overset{n-1}{\underset{l=2}{⋃}}M\left(s,l\right)|\\ \; & \underset{=}{\left(c\right)}|M(s,2)|. \end{array}$$

The first equality (a) in (50) is derived by using $M^{m}\left(s,l\right)\cap M^{m}\left(s,l^{\prime }\right)=\emptyset$ for all $l\neq l^{\prime }$. The second inequality (b) is derived by using $M^{m}\left(s,l\right)\subseteq M\left(s,l\right)$. The third equality (c) is derived by using $M\left(s,l^{\prime }\right)\subseteq M\left(s,l\right)$ for all $l^{\prime }>l$.

Therefore, if $|M^{*}\left(s_{0},l_{0}\right)|>|M\left(s,2\right)|$ for any $s\neq s_{0}$, the first stage of the blind reconstruction method of BCH codes in [15] always succeeds. Furthermore, by the Equations (49) and (50), the inequality (48) clearly holds. □

If the first stage succeeds, in order for the second stage to succeed, the following relation should be satisfied,

(51) $$|M^{m}\left(s_{0},l_{0}\right)\left|>\right|M^{m}\left(s_{0},l\right)|,\forall l\neq l_{0}.$$

The relation (51) can be simplified as in the following Lemma 6.

**Lemma** **6.** If $|M^{*}\left(s_{0},l_{0}\right)|>|M\left(s,2\right)|,\forall s\neq s_{0}$, holds and the following inequality is satisfied, then the second stage of the blind reconstruction of BCH codes in [15] always succeeds,

(52) $$|M^{*}\left(s_{0},l_{0}\right)\left|>\right|M^{e}\left(s_{0},2\right)|,$$

*and the success probability of the second stage is lower-bounded as*

(53) $$\mathrm{Pr}\left(|M^{m}\left(s_{0},l_{0}\right)\left|>\right|M^{m}\left(s_{0},l\right)|,\forall l\neq l_{0}\right)\geq \mathrm{Pr}\left(|M^{*}\left(s_{0},l_{0}\right)\left|>\right|M^{e}\left(s_{0},2\right)|\right),$$

where, for better readability, the given condition that $|M^{*}\left(s_{0},l_{0}\right)|>|M\left(s,2\right)|,\forall s\neq s_{0}$, is omitted in the probability.

**Proof.**  Since $|M^{*}\left(s_{0},l_{0}\right)|>|M\left(s,2\right)|$ for any $s\neq s_{0}$, $|M^{m}\left(s_{0},l_{0}\right)\left|>\right|M^{m}\left(s_{0},l\right)|$ holds for $l>l_{0}$ as follows:

(54) $$\begin{array}{cc} |M^{m}\left(s_{0},l\right)| & \underset{\leq }{\left(a\right)}|M\left(s_{0}+1,l-1\right)|\\ \; & \underset{<}{\left(b\right)}|M^{*}\left(s_{0},l_{0}\right)|\\ \; & \underset{\leq }{\left(c\right)}|M^{m}\left(s_{0},l_{0}\right)|. \end{array}$$

The inequality (a) in (54) is derived by using $M^{m}\left(s_{0},l\right)\subseteq M\left(s_{0}+1,l-1\right)$. The inequality (b) is derived by using $|M^{*}\left(s_{0},l_{0}\right)|>|M\left(s,2\right)|$ for $s\neq s_{0}$ and $|M\left(s_{0}+1,2\right)|\geq |M\left(s_{0}+1,l-1\right)|$. The inequality (c) is derived by using $M^{*}\left(s_{0},l_{0}\right)\subseteq M^{m}\left(s_{0},l_{0}\right)$. Therefore, it remains to prove that our assumption implies $|M^{m}\left(s_{0},l_{0}\right)\left|>\right|M^{m}\left(s_{0},l\right)|$ for $l<l_{0}$.

If the *j*-th received codeword $r_{j}\left(x\right)$ is error-free (i.e., $e_{j}\left(x\right)=0$), then $r_{j}\left(x\right)\notin M^{m}\left(s_{0},l\right)$ for all $l<l_{0}$ because $r_{j}\left(x\right)\in M\left(s_{0},l_{0}\right)$ always holds. Therefore, $e_{j}\left(x\right)\neq 0$ for $r_{j}\left(x\right)\in M^{m}\left(s_{0},l\right)$, $l<l_{0}$ and then, the relation (51) can be simplified as $|M^{m}\left(s_{0},l_{0}\right)\left|>\right|M^{m}\left(s_{0},l\right)\cap \left\{r_{j}\left(x\right)|e_{j}\left(x\right)\neq 0\right\}|$ for $l<l_{0}$. Since it is always satisfied that $|M^{m}\left(s_{0},l\right)\cap \left\{r_{j}\left(x\right)|e_{j}\left(x\right)\neq 0\right\}|\leq |M\left(s_{0},l\right)\cap \left\{r_{j}\left(x\right)|e_{j}\left(x\right)\neq 0\right\}\left|=\right|M^{e}\left(s_{0},l\right)|$, if $|M^{m}\left(s_{0},l_{0}\right)\left|>\right|M^{e}\left(s_{0},l\right)|$ for $l<l_{0}$, the second stage succeeds. Furthermore, since $|M^{e}\left(s_{0},l\right)\left|>\right|M^{e}\left(s_{0},l^{\prime }\right)|$ for $l<l^{\prime }$, if $|M^{m}\left(s_{0},l_{0}\right)\left|>\right|M^{e}\left(s_{0},2\right)|$ is satisfied, then the second stage also succeeds. Note that the LHS in (51) satisfies that $|M^{m}\left(s_{0},l_{0}\right)\left|\geq \right|M^{*}\left(s_{0},l_{0}\right)|$. Therefore, if $|M^{*}\left(s_{0},l_{0}\right)\left|\geq \right|M^{e}\left(s_{0},2\right)|$ is satisfied, then the second stage always succeeds.

The lower-bound on the success probability of the second stage is derived as follows:

(55) $$\begin{array}{cc} \; & \mathrm{Pr}\left(|M^{m}\left(s_{0},l_{0}\right)\left|>\right|M^{m}\left(s_{0},l\right)|,\forall l\neq l_{0}\right)\\ \; & =\mathrm{Pr}\left(|M^{m}\left(s_{0},l_{0}\right)\left|>\right|M^{m}\left(s_{0},l\right)|,\forall l\leq l_{0}\right)\\ \; & =\mathrm{Pr}\left(|M^{m}\left(s_{0},l_{0}\right)\left|>\right|M^{m}\left(s_{0},l\right)\cap \left\{r_{j}\left(x\right)|e_{j}\left(x\right)\neq 0\right\}|,\forall l\leq l_{0}\right)\\ \; & \geq \mathrm{Pr}\left(|M^{m}\left(s_{0},l_{0}\right)|>|M\left(s_{0},l\right)\cap \left\{r_{j}\left(x\right)|e_{j}\left(x\right)\neq 0\right\}|,\forall l\leq l_{0}\right)\\ \; & =\mathrm{Pr}\left(|M^{m}\left(s_{0},l_{0}\right)\left|>\right|M^{e}\left(s_{0},l\right)|,\forall l\leq l_{0}\right)\\ \; & =\mathrm{Pr}\left(|M^{m}\left(s_{0},l_{0}\right)\left|>\right|M^{e}\left(s_{0},2\right)|\right)\\ \; & \geq \mathrm{Pr}\left(|M^{*}\left(s_{0},l_{0}\right)\left|>\right|M^{e}\left(s_{0},2\right)|\right). \end{array}$$

Note that, for better readability, the given condition that $|M^{*}\left(s_{0},l_{0}\right)|>|M\left(s,2\right)|,\forall s\neq s_{0}$, is omitted in the probability. □

By Lemmas 5 and 6, if $|M^{*}\left(s_{0},l_{0}\right)|>\max_{s\neq s_{0}}\left|M\left(s,2\right)\right|$ and $|M^{*}\left(s_{0},l_{0}\right)\left|>\right|M^{e}\left(s_{0},2\right)|$, then the blind reconstruction method of BCH code in [15] always succeeds. Moreover, the success probability of the blind reconstruction method of BCH code in [15] is lower-bounded, as in the following Theorem 1.

**Theorem** **1.** *Suppose that randomly generated M codewords of $BCH_{q}\left(n,k\right)$, which uses the generator polynomial g(x) as in (1) with $b=s_{0}$ and $d=l_{0}+1$, are received after passing through q-ary symmetric channel with error probability ϵ. Then, the success probability of the blind reconstruction method of BCH codes in [15], denoted as $P_{s}$, is lower-bounded as follows:*

(56) $$P_{s}\geq \sum_{x=1}^{M}B\left(M,x,p_{0}\right)\prod_{C_{i}\subseteq {C_{s_{0}}^{l_{0}}}^{c}}\left\{\sum_{y=0}^{x-1}B\left(M,y,\frac{1}{q^{rk(\alpha^{i})}}\right)\right\}\prod_{C_{j}\subseteq C_{s_{0}}^{l_{0}}}\left\{\sum_{y=0}^{x-1}B\left(M,y,P_{ue}\left(C_{j}\right)\right)\right\},$$

*where B(M,x,p)=Mxpx(1−p)M−x, $p_{0}=\left\{{\left(1-\epsilon \right)}^{n}+P_{ue}\left(C_{s_{0}}^{l_{0}}\right)\right\}\prod_{z\in {C_{s_{0}}^{l_{0}}}^{c}}\left(1-1/q^{rk(\alpha^{z})}\right)$, ${C_{s_{0}}^{l_{0}}}^{c}=\left\{1,2,\cdots ,n-1\right\}\setminus C_{s_{0}}^{l_{0}}$, and $P_{ue}\left(C\right)$ is the undetectable error probability of BCH code having $\left\{\alpha^{i}|i\in C\right\}$ as its null spectrum.*

**Proof.**  By Lemmas 5 and 6, if $|M^{*}\left(s_{0},l_{0}\right)|>\max_{s\neq s_{0}}\left|M\left(s,2\right)\right|$ and $|M^{*}\left(s_{0},l_{0}\right)\left|>\right|M^{e}\left(s_{0},2\right)|$, then the blind reconstruction of BCH codes in [15] always succeeds. In order to calculate the probability that $|M^{*}\left(s_{0},l_{0}\right)|>\max_{s\neq s_{0}}\left|M\left(s,2\right)\right|$ and $|M^{*}\left(s_{0},l_{0}\right)\left|>\right|M^{e}\left(s_{0},2\right)|$, the probabilities that $r_{j}\left(x\right)\in M^{*}\left(s_{0},l_{0}\right)$, $r_{j}\left(x\right)\in M\left(s,2\right)$, and $r_{j}\left(x\right)\in M^{e}\left(s_{0},2\right)$ should be calculated, respectively.

If the *j*-th received codeword $r_{j}\left(x\right)$ is error-free or has an undetectable error, it is always true that $r_{j}\left(x\right)\in M\left(s_{0},l_{0}\right)$. Furthermore, if $r_{j}\left(\alpha^{i}\right)\neq 0$ for $i\in {C_{s_{0}}^{l_{0}}}^{c}$, it is also true that $r_{j}\left(x\right)\in M^{*}\left(s_{0},l_{0}\right)$, where ${C_{s_{0}}^{l_{0}}}^{c}=\left\{1,2,\cdots ,n-1\right\}\setminus C_{s_{0}}^{l_{0}}$. Then, $\mathrm{Pr}\left(r_{j}\left(x\right)\in M^{*}\left(s_{0},l_{0}\right)\right)$ is derived as follows:

(57) $$\begin{array}{cc} \mathrm{Pr}\left(r_{j}\left(x\right)\in M^{*}\left(s_{0},l_{0}\right)\right) & =\mathrm{Pr}\left(r_{j}\left(\alpha^{i}\right)=0,\forall i\in C_{s_{0}}^{l_{0}},r_{j}\left(\alpha^{z}\right)\neq 0,\forall z\in {C_{s_{0}}^{l_{0}}}^{c}\right)\\ \; & \underset{=}{\left(a\right)}\mathrm{Pr}\left(r_{j}\left(\alpha^{i}\right)=0,\forall i\in C_{s_{0}}^{l_{0}}\right)\prod_{z\in {C_{s_{0}}^{l_{0}}}^{c}}\mathrm{Pr}\left(r_{j}\left(\alpha^{z}\right)\neq 0\right)\\ \; & \underset{=}{\left(b\right)}\left\{{\left(1-\epsilon \right)}^{n}+P_{ue}\left(C_{s_{0}}^{l_{0}}\right)\right\}\prod_{z\in {C_{s_{0}}^{l_{0}}}^{c}}\left(1-\frac{1}{q^{rk\left(\alpha^{z}\right)}}\right)\\ \; & ≜p_{0}, \end{array}$$

where $P_{ue}\left(C_{s_{0}}^{l_{0}}\right)$ is the undetectable error probability of BCH code having $\left\{\alpha^{i}|i\in C_{s_{0}}^{l_{0}}\right\}$ as its null spectrum. In the equality (a) in (57), $r_{j}\left(\alpha^{i}\right)$ for $i\in {C_{s_{0}}^{l_{0}}}^{c}$ occurs uniformly at random because the message is generated uniformly at random. Therefore, the event that $r_{j}\left(\alpha^{i}\right)=0$ for any $i\in C_{s_{0}}^{l_{0}}$ and the event that $r_{j}\left(\alpha^{z}\right)=0$ for any $z\in {C_{s_{0}}^{l_{0}}}^{c}$ are independent and hence the equality (a) holds. The equality (b) is derived by using $\mathrm{Pr}\left(r_{j}\left(\alpha^{i}\right)=0,\forall i\in C_{s_{0}}^{l_{0}}\right)=\left\{{\left(1-\epsilon \right)}^{n}+P_{ue}\left(C_{s_{0}}^{l_{0}}\right)\right\}$ and Lemma 4.

The probability that $r_{j}\left(x\right)\in M\left(s,2\right)$ for $s\neq s_{0}$ is calculated by using Lemma 4 as follows:

(58) $$\begin{array}{cc} \mathrm{Pr}\left(r_{j}\left(x\right)\in M\left(s,2\right)\right) & =\mathrm{Pr}\left(r_{j}\left(\alpha^{i}\right)=0,\forall i\in C_{s}^{2}\right)\\ \; & =\mathrm{Pr}\left(r_{j}\left(\alpha^{i}\right)=0,\forall i\in C_{s}^{2}\cap C_{s_{0}}^{l_{0}}\right)\mathrm{Pr}\left(r_{j}\left(\alpha^{z}\right)=0,\forall z\in C_{s}^{2}\setminus C_{s_{0}}^{l_{0}}\right)\\ \; & =\mathrm{Pr}\left(r_{j}\left(\alpha^{i}\right)=0,\forall i\in C_{s}^{2}\cap C_{s_{0}}^{l_{0}}\right)\prod_{z\in C_{s}^{2}\setminus C_{s_{0}}^{l_{0}}}\mathrm{Pr}\left(r_{j}\left(\alpha^{z}\right)=0\right)\\ \; & =P_{ue}\left(C_{s}^{2}\cap C_{s_{0}}^{l_{0}}\right)\prod_{z\in C_{s}^{2}\setminus C_{s_{0}}^{l_{0}}}\frac{1}{q^{rk(\alpha^{z})}}. \end{array}$$

For better readability, $s\neq s_{0}$ is omitted in the probability.

Let $M_{1}\left(C_{i}\right)$ be $\left\{r_{j}\left(x\right)|r_{j}\left(\alpha^{z}\right)=0,\forall z\in C_{i}\right\}$ and $M_{2}\left(C_{i}\right)$ be $\left\{r_{j}\left(x\right)|r_{j}\left(\alpha^{z}\right)=c_{j}\left(\alpha^{z}\right)+e_{j}\left(\alpha^{z}\right)=0,\forall z\in C_{i},e_{j}\left(x\right)\neq 0\right\}$. If $|M^{*}\left(s_{0},l_{0}\right)|$ is greater than $|M_{1}\left(C_{i}\right)|$ for any $C_{i}\subseteq {C_{s_{0}}^{l_{0}}}^{c}$ and also greater than $|M_{2}\left(C_{i}\right)|$ for any $C_{i}\subseteq C_{s_{0}}^{l_{0}}$, it is also satisfied that $|M^{*}\left(s_{0},l_{0}\right)|>|M\left(s,2\right)|$ for any $s\neq s_{0}$. It is because if there exists $C_{i}\subseteq C_{s}^{2}$ such that $C_{i}\subseteq {C_{s_{0}}^{l_{0}}}^{c}$, it is true that $|M^{*}\left(s_{0},l_{0}\right)|>|M\left(s,2\right)|$ due to $|M^{*}\left(s_{0},l_{0}\right)\left|>\right|M_{1}\left(C_{i}\right)|\geq |M\left(s,2\right)|$ for any $C_{i}\subseteq {C_{s_{0}}^{l_{0}}}^{c}$. Furthermore, if there exists $C_{i}\subseteq C_{s}^{2}$ such that $C_{i}\subseteq C_{s_{0}}^{l_{0}}$, then it is also true that $|M^{*}\left(s_{0},l_{0}\right)|>|M\left(s,2\right)|$ due to $|M^{*}\left(s_{0},l_{0}\right)\left|>\right|M_{2}\left(C_{i}\right)|\geq |M\left(s,2\right)|$ for any $C_{i}\subseteq C_{s_{0}}^{l_{0}}$. Then, the condition for the success of the first stage of blind reconstruction method is simplified as follows:

(59) $$|M^{*}\left(s_{0},l_{0}\right)\left|>\right|M_{1}\left(C_{i}\right)\left|,\right|M^{*}\left(s_{0},l_{0}\right)\left|>\right|M_{2}\left(C_{j}\right)|,\forall C_{i}\subseteq {C_{s_{0}}^{l_{0}}}^{c},\forall C_{j}\subseteq C_{s_{0}}^{l_{0}}.$$

Moreover, $\mathrm{Pr}(r_{j}\left(\alpha^{z}\right)=0,\forall z\in C_{i})$ for $C_{i}\subseteq {C_{s_{0}}^{l_{0}}}^{c}$ is simplified as follows:

(60) $$\begin{array}{cc} \mathrm{Pr}\left(r_{j}\left(\alpha^{z}\right)=0,\forall z\in C_{i}\subseteq {C_{s_{0}}^{l_{0}}}^{c}\right) & =\mathrm{Pr}\left(r_{j}\left(x\right)\in M\left(i,1\right),C_{i}\subseteq {C_{s_{0}}^{l_{0}}}^{c}\right)\\ \; & \underset{=}{\left(a\right)}P_{ue}\left(C_{i}\cap C_{s_{0}}^{l_{0}}\right)\prod_{C_{j}\subseteq C_{i}\setminus C_{s_{0}}^{l_{0}}}\frac{1}{q^{rk(\alpha^{j})}}\\ \; & \underset{=}{\left(b\right)}\frac{1}{q^{rk(\alpha^{i})}}. \end{array}$$

The equality (a) is derived by using (58) and (b) is derived by using $C_{i}\cap C_{s_{0}}^{l_{0}}=\emptyset$ and $C_{i}\setminus C_{s_{0}}^{l_{0}}=C_{i}$. Furthermore, $\mathrm{Pr}(r_{j}\left(\alpha^{z}\right)=0,\forall z\in C_{i},e_{j}\left(x\right)\neq 0)$ for $C_{i}\subseteq C_{s_{0}}^{l_{0}}$, is also simplified as follows:

(61) $$\begin{array}{cc} \mathrm{Pr}\left(r_{j}\left(\alpha^{z}\right)=0,\forall z\in C_{i}\subseteq C_{s_{0}}^{l_{0}},e_{j}\left(x\right)\neq 0\right) & =\mathrm{Pr}\left(r_{j}\left(x\right)\in M\left(i,1\right),C_{i}\subseteq C_{s_{0}}^{l_{0}}\right)\\ \; & =P_{ue}\left(C_{i}\cap C_{s_{0}}^{l_{0}}\right)\prod_{C_{j}\subseteq C_{i}\setminus C_{s_{0}}^{l_{0}}}\frac{1}{q^{rk(\alpha^{j})}}\\ \; & \underset{=}{\left(a\right)}P_{ue}\left(C_{i}\right). \end{array}$$

The equality (a) is derived by using $C_{i}\cap C_{s_{0}}^{l_{0}}=C_{i}$ and $C_{i}\setminus C_{s_{0}}^{l_{0}}=\emptyset$

The probability that $r_{j}\left(x\right)\in M^{e}\left(s_{0},2\right)$ is the same as the undetectable error probability of a BCH code having $\left\{\alpha^{i}|i\in C_{s_{0}}^{2}\right\}$ as its null spectrum as follows:

(62) $$\begin{array}{c} \mathrm{Pr}(r_{j}\left(x\right)\in M^{e}\left(s_{0},2\right))=P_{ue}\left(C_{s_{0}}^{2}\right). \end{array}$$

If $|M^{*}\left(s_{0},l_{0}\right)\left|>\right|M_{2}\left(C_{i}\right)|$ for $C_{i}\subseteq C_{s_{0}}^{2}$, then the second stage of the blind reconstruction method succeeds. Therefore, $\mathrm{Pr}(|M^{*}\left(s_{0},l_{0}\right)\left|>\right|M^{e}\left(s_{0},2\right)\left|\right)\geq \mathrm{Pr}(|M^{*}\left(s_{0},l_{0}\right)\left|>\right|M_{2}\left(C_{i}\right)\left|\right)$ for $C_{i}\subseteq C_{s_{0}}^{2}$.

Finally, by using (57)–(62), $P_{s}$ is lower-bounded as

(63) $$\begin{array}{cc} P_{s} & \geq \mathrm{Pr}\left(|M^{*}\left(s_{0},l_{0}\right)|>\max_{s\neq s_{0}}|M\left(s,2\right)\left|,\right|M^{*}\left(s_{0},l_{0}\right)\left|>\right|M^{e}\left(s_{0},2\right)|\right)\\ \; & =\sum_{x=1}^{M}\mathrm{Pr}\left(|M^{*}\left(s_{0},l_{0}\right)|=x\right)\mathrm{Pr}\left(\max_{s\neq s_{0}}\left|M\left(s,2\right)|<x,\right|M^{e}\left(s_{0},2\right)\left|<x\;|\;\right|M^{*}\left(s_{0},l_{0}\right)|=x\right)\\ \; & \underset{\geq }{\left(a\right)}\sum_{x=1}^{M}\mathrm{Pr}\left(|M^{*}\left(s_{0},l_{0}\right)|=x\right)\prod_{C_{i}\subseteq {C_{s_{0}}^{l_{0}}}^{c}}\mathrm{Pr}\left(|M_{1}\left(C_{i}\right)|<x\right)\prod_{C_{j}\subseteq C_{s_{0}}^{l_{0}}}\mathrm{Pr}\left(|M_{2}\left(C_{j}\right)|<x\right)\\ \; & =\sum_{x=1}^{M}B\left(M,x,p_{0}\right)\prod_{C_{i}\subseteq {C_{s_{0}}^{l_{0}}}^{c}}\left\{\sum_{y=0}^{x-1}B\left(M,y,\frac{1}{q^{rk(\alpha^{i})}}\right)\right\}\prod_{C_{j}\subseteq C_{s_{0}}^{l_{0}}}\left\{\sum_{y=0}^{x-1}B\left(M,y,P_{ue}\left(C_{j}\right)\right)\right\}. \end{array}$$

The inequality (a) in (63) is derived by using $\mathrm{Pr}(|M^{*}\left(s_{0},l_{0}\right)\left|>\right|M^{e}\left(s_{0},2\right)\left|\right)\geq \mathrm{Pr}(|M^{*}\left(s_{0},l_{0}\right)\left|>\right|M_{2}\left(C_{i}\right)\left|\right)$ for $C_{i}\subseteq C_{s_{0}}^{2}$. Note that the event of $|M_{z}\left(C_{i}\right)|<x$ is independent with the event of $|M_{z}\left(C_{j}\right)|<x$ for i≠j and z∈{1,2} because $C_{i}\cap C_{j}=\emptyset$ for i≠j. Furthermore, the event of $|M_{1}\left(C_{i}\right)|<x$ and the event of $|M_{2}\left(C_{j}\right)|<x$ for i≠j are also independent because $C_{i}\cap C_{j}=\emptyset$ for i≠j. □

In Theorem 1, a lower-bound on the success probability of the blind reconstruction method of BCH codes in [15] is obtained. In order to confirm the validity of this lower-bound, simulations are performed by using the following BCH codes.

- $BCH_{2}\left(31,21\right)$, $BCH_{2}\left(63,51\right)$, $BCH_{2}\left(127,113\right)$: These are binary BCH codes having $\left\{\alpha^{i}|i\in C_{1}^{4}\right\}$ as their null spectrum.
- $BCH_{32}\left(31,29\right)$, $BCH_{64}\left(63,59\right)$: These are Reed–Solomon (RS) codes having $\left\{\alpha^{i}|i\in C_{1}^{4}\right\}$ as their null spectrum.

**Figure 1:**
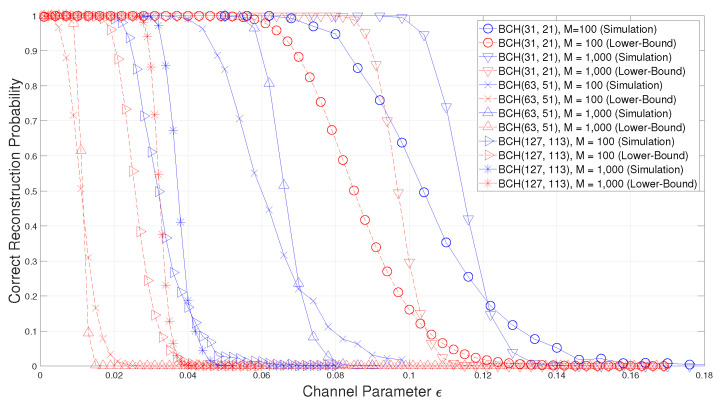
Comparison of the correct reconstruction probability with the proposed lower-bound for binary Bose–Chaudhuri–Hocquenghem (BCH) codes.

As you can see from Figure 1, the success probability of the blind reconstruction of binary BCH codes is well bounded by the lower-bound in (56). However, for $BCH_{2}\left(63,51\right)$, the gap between the simulation result and the lower-bound is larger than the others because $BCH_{2}\left(63,51\right)$ has a cyclotomic coset of cardinality 2, while all the cyclotomic cosets of $BCH_{2}\left(31,21\right)$ and $BCH_{2}\left(127,113\right)$ have the cardinality 5 and 7, respectively. In (56), if a cyclotomic coset $C_{i}\subseteq {C_{s_{0}}^{l_{0}}}^{c}$ has small cardinality, $1/q^{rk(\alpha^{i})}$ becomes bigger and then, $B(M,y,1/q^{rk(\alpha^{i})})$ becomes smaller. Therefore, the lower-bounds of the blind reconstruction performance of $BCH_{2}\left(31,21\right)$ and $BCH_{2}\left(127,113\right)$ is much tighter than $BCH_{2}\left(63,51\right)$.

**Figure 2:**
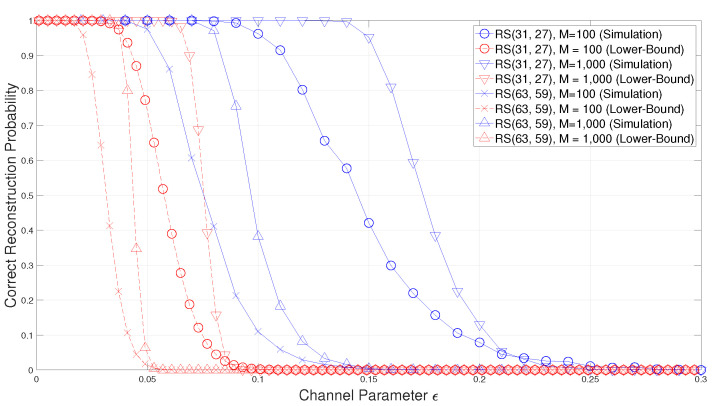
Comparison of the correct reconstruction probability with the proposed lower-bound for Reed–Solomon (RS) codes.

As you can see from Figure 2, the success probability of the blind reconstruction of RS codes is also well bounded by the lower-bound in (56). Moreover, as the code length increase, the proposed lower-bound of RS codes becomes tighter and therefore this lower-bound can be a good estimation of blind reconstruction performance for practical RS codes. Furthermore, since the proposed lower-bound can estimate the blind reconstruction performance without the extensive simulation, the proposed lower-bound is suitable for practical use.

## 5. Conclusions

The blind reconstruction method of BCH codes in [15] shows the best performance, but the theoretical analysis of this method has not been performed. In this paper, by analyzing the properties of BCH codes on the aspects of blind reconstruction, a lower-bound on the success probability of the blind reconstruction method in [15] is derived. Especially, the distribution of GFFT values of the received codewords are analyzed and the blind reconstruction method is formalized based on the conjugacy classes. Furthermore, the analysis results can be applied not only to the binary BCH codes, but also to the non-binary BCH codes, including RS codes. By comparing the derived lower-bound with the simulation results, it is confirmed that the success probability of the blind reconstruction is well bounded by the proposed lower-bound.
